# Risk factors for mortality in surgical patients on combined continuous renal replacement therapy and extracorporeal membrane oxygenation: single-center retrospective study

**DOI:** 10.1080/0886022X.2023.2282019

**Published:** 2023-11-20

**Authors:** Suiqing Huang, Junjie Wang, Kangni Feng, Huawei Wu, Liqun Shang, Yang Huang, Zhuoming Zhou, Huayang Li, Quan Liu, Jiantao Chen, Mengya Liang, Jian Hou, Guangxian Chen, Zhongkai Wu

**Affiliations:** a Department of Cardiac Surgery, First Affiliated Hospital of Sun Yat-sen University, Guangzhou, China; b Department of Epidemiology, Mailman School of Public Health, Columbia University, New York, NY, USA; c Department of Cardiology, Guangzhou Panyu Central Hospital, Guangzhou, China; d Department of Cardiothoracic Surgery ICU, First Affiliated Hospital of Sun Yat-sen University, Guangzhou, China

**Keywords:** Extracorporeal membrane oxygenation, continuous renal replacement therapy, acute renal injury, risk factors, surgery, mortality

## Abstract

**Objective:**

In patients receiving extracorporeal membrane oxygenation (ECMO), continuous renal replacement therapy (CRRT) is increasingly being used for renal replacement and fluid management. However, critically ill surgical patients receiving combined ECMO and CRRT tend to have a high mortality rate, and there are limited studies on this population. Therefore, we aimed to investigate the risk factors for mortality in surgical patients receiving combined ECMO and CRRT.

**Methods:**

Data of surgical patients who underwent ECMO between December 2013 and April 2023 were retrospectively reviewed. Univariate and multivariate logistic regression analysis were used to identify the risk variables. Receiver operating characteristic (ROC) curve analysis was used to determine the cutoff value of albumin and age to predict death.

**Results:**

A total of 199 patients on ECMO support were screened, of which 105 patients were included in the final analysis. Of 105 patients, 77 (73.33%) were treated with CRRT. Veno-arterial ECMO was performed in 97 cases (92.38%), and the rest were veno-venous ECMO (*n* = 8, 7.62%). Cardiovascular-related surgery was performed in the main patients (*n* = 86, 81.90%) and other types of surgery in 19 patients. In surgical patients on ECMO support, the logistic regression analysis showed that CRRT implantation, male sex, and age were the independent risks factors for mortality. Furthermore, the ROC curve analysis showed that age 48.5 years had the highest Youden index. In surgical patients on combined CRRT and ECMO, age, valvular heart disease, and albumin were the independent risk factors for prognosis. Albumin had the highest Youden index at a cutoff value of 39.95 g/L for predicting mortality, though the overall predictive value was modest (area under ROC 0.704). Age had the highest Youden index at a cutoff value of 48.5 years for predicting mortality.

**Conclusions:**

In our cohort of surgical patients requiring ECMO, which consisted mostly of patients undergoing cardiovascular surgery requiring VA-ECMO, the need for CRRT was an independent risk factor for mortality. In the subset of patients on combined CRRT and ECMO, independent risk factors for mortality included higher age, lack of valvular heart disease, and lower serum albumin.

## Introduction

Extracorporeal membrane oxygenation (ECMO) is a life-saving treatment for patients with serious, life-threatening heart and lung dysfunction by temporarily bypassing the functions of these organs. However, patients receiving ECMO have a significant risk of developing multiple organ failures and related complications, such as acute kidney injury (AKI) and fluid overload [1].

Reportedly, AKI is a common indication for approximately 42%–85% of patients receiving ECMO [2—4]. The pathogenesis and high frequency of AKI in ECMO patients are intrinsically complicated, owing to the underlying illness and other ECMO-related variables [4,5]. In these cases, continuous renal replacement therapy (CRRT) is increasingly being used for renal replacement and fluid management. In critically ill patients, the mortality rate for AKI was estimated to be 40%–70% [6,7], and the mortality rate for ECMO-related AKI was as high as 80% [8]. In clinical practice, we found that surgical patients requiring ECMO implantation, especially after cardiac surgery, often also require CRRT, and the condition of these patients is often more severe, with a high mortality rate. Recent studies on combined ECMO and CRRT have mainly focused on medical patients [9–11], especially those with coronavirus disease 2019 [12,13]. Studies on combined ECMO and CRRT in surgical patients are scarce, with the exception of the only study with children as the study population by Gupta et al. [14]. Faced with the clinical dilemma of high mortality and the difficult and tricky management of critically ill surgical patients, there is an urgent need to focus and conduct studies on this population.

Therefore, in this study, we retrospectively analyzed the surgical patients on ECMO at our center in the past approximately 9 years. We aimed to investigate the independent risk factors for mortality in patients on combined CRRT and ECMO, which can help us find breakthroughs for high mortality and focus on the modifiable risk factors.

## Patients and methods

### Study participants and data collection

**Figure 1.**
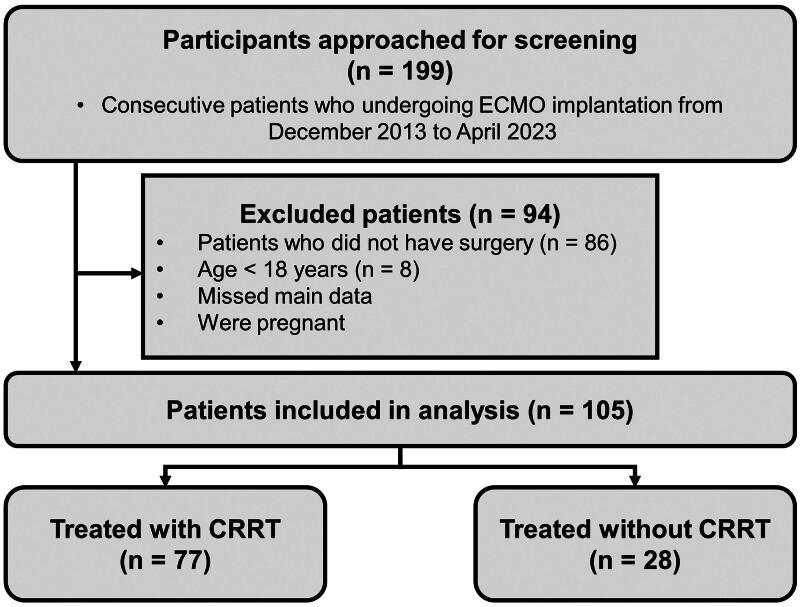
Flow chart for study inclusion/exclusion of patients with ECMO implication. ECMO: extracorporeal membrane oxygenation; CRRT: continuous renal replacement therapy.

We searched the patient database of the First Affiliated Hospital of Sun Yat-sen University and selected 199 consecutive patients who underwent ECMO implantation between December 2013 and April 2023. The exclusion criteria were as follows: (1) no surgery, (2) age <18 years, (3) missing data, and (4) pregnancy. Finally, 105 patients who underwent surgery and were treated with ECMO were included. Of 105 patients, 77 were treated with CRRT (Figure 1). Clinical data including demographic factors, medical history, clinical characteristics, surgical details, and ancillary tests were obtained from the patients’ medical records. The study was reviewed and approved by the Institutional Review Board of the First Affiliated Hospital of Sun Yat-sen University (approval number: 2011 [31]) and in accordance with the Helsinki Declaration of 1975.

### Study definitions and outcome

In this study, the follow-up time to determine the end point of death was during the patient’s hospitalization or within 30 d after surgery. ECMO was all implanted preoperatively, intraoperatively or postoperatively, and CRRT could start preoperatively or start postoperatively, but there was some overlap in the use of time with ECMO. The outcome then occurred after the concomitant use of ECMO and CRRT. The indication for VA-ECMO implantation is patients with Interagency Registry for Mechanically Assisted Circulatory Support (INTERMACS) profile I or II [15]. Indications for the use of VV-ECMO are the same as the Extracorporeal Life Support Organization (ELSO) guidelines [16]. Current heart failure was defined as New York Heart Association class III/IV at enrollment. A history of prior cardiac surgery was defined as open-heart cardiac surgery and does not include interventional procedures. Chronic kidney disease was defined as GFR categories G3a–G5 according to the Kidney Disease: Improving Global Outcomes (KDIGO) criteria [17]. The diagnosis and staging of AKI is based on the KDIGO guidelines [18]. The blood test result was based on the last test result before the surgery. Other variables and cutoffs were defined using the International Classification of Diseases, Ninth Revision, Clinical Modification (ICD9CM) codes unless otherwise stated.

### Statistical analysis

According to normality, continuous variables were reported as mean ± standard deviation (SD) or medians (interquartile range) and were compared using the unpaired Student t-test or Mann–Whitney U test, as applicable. The chi-squared or Fisher exact test was used to compare categorical variables. The risk variables were identified using univariate and multivariate logistic regression analysis. The variables for multivariate regression are based on variables with a P value < 0.1 under univariate results and the step method was used for the multivariate regression. The receiver operating characteristic (ROC) curve analysis was used to assess the predictive ability and determine the cutoff values. All tests were two-tailed, and *p <* .05 was considered statistically significant. All statistical analyses were performed using SPSS version 26.0 software (SPSS Inc., Chicago, IL).

## Results

### Patient characteristics

Preliminary screening identified 199 patients treated with ECMO implantations between December 2013 and April 2023. Of them, only 105 patients were included in the final analysis (Figure 1). Of 105, 83 (79.05%) patients died in the hospital or within 30 d after surgery. The baseline characteristics of the patients are presented in Table 1. The median age of the patients was 57 years (range, 47–63.5), with men accounting for more than two-thirds (69.52%) of the entire study population. Heart failure occurred in 88 (83.81%) patients, and atrial fibrillation/flutter occurred in 22 (20.95%) patients. A total of 43 (40.95%) patients had a history of previous surgery, of whom 16 (15.24%) had a history of previous cardiac surgery. In addition, 21 (20%) patients were diagnosed with coronary artery disease, 37 (35.24%) with valvular disease, and 11 (10.48%) with congenital heart disease. Compared to those who died, patients who survived were older and had significantly lower lymphocyte counts and aspartate aminotransferase levels (all *p* < .05). It can be observed that the difference in AKI stage between the survival and death patient groups is not statistically significant.

**Table 1.** Demographic and clinical characteristics of surgical patients receiving ECMO support.

Variables	Total (*n* = 105)	Survival (*n* = 22)	Death (*n* = 83)	*p*-value
Age (years)	57 (47–63.5)	50 (40.25–58.5)	57 (49–64)	**.039** *
Sex, male	73 (69.52)	19 (86.36)	54 (65.06)	.054
Medical history				
Hypertension	42 (40)	7 (31.82)	35 (42.17)	.378
Diabetes mellitus	13 (12.38)	2 (9.09)	11 (13.25)	.598
Previous PCI	7 (6.67)	1 (4.55)	6 (7.23)	.654
Previous MI	8 (7.62)	2 (9.09)	6 (7.23)	.770
COPD	1 (0.95)	0	1 (1.20)	.605
Renal insufficiency	7 (6.67)	1 (4.55)	6 (7.23)	.654
Cerebrovascular disease	8 (7.62)	2 (9.09)	6 (7.23)	.770
Tumor history	11 (10.48)	1 (4.55)	10 (12.05)	.307
Smoking	24 (22.86)	7 (31.82)	17 (20.48)	.260
Heart failure	88 (83.81)	21 (95.45)	67 (80.72)	.095
Atrial fibrillation/flutter	22 (20.95)	4 (18.18)	18 (21.69)	.719
Previous surgical history	43 (40.95)	6 (27.27)	37 (44.58)	.142
Previous cardiac surgery	16 (15.24)	2 (9.09)	14 (16.87)	.367
Coronary heart disease	21 (20.00)	3 (13.64)	18 (21.69)	.401
Valvular disease	37 (35.24)	9 (40.91)	28 (33.73)	.531
Congenital cardiac defects	11 (10.48)	4 (18.18)	7 (8.43)	.184
Laboratory data				
White blood cells (×10^9^/L)	7.45 (5.59–9.79)	8.64 (6.55–11.07)	7.11 (5.17–9.71)	.141
Hemoglobin (g/L)	132 (119–149.5)	134 (122–153)	132 (119–149)	.524
Platelets (×10^9^/L)	197 (140.5–230.5)	208.5 (161.75–280)	192 (135–227)	.190
Lymphocytes (×10^9^/L)	1.47 (1.22–2.04)	1.80 (1.39–2.19)	1.42 (1.15–1.99)	**.042** *
Monocytes (×10^9^/L)	0.53 (0.40–0.74)	0.63 (0.52–0.75)	0.51 (0.37–0.74)	.052
ALT (U/L)	25 (16.5–42)	34 (22.5–51.25)	24 (16–40)	**.037** *
AST (U/L)	29 (23–44)	33 (26–54.5)	29 (22–44)	.212
Albumin (g/L)	37.35 ± 4.82	39 ± 6.44	36.91 ± 4.23	.071
Total bilirubin (umol/L)	18 (12.25–28.25)	15.65 (12.15–22.40)	18.3 (12.2–31.2)	.321
Creatinine (umol/L)	85 (72.5–112)	85.5 (69.75–106.25)	85 (73–112)	.912
Urea nitrogen (mmol/L)	6.4 (4.8–9.7)	6.35 (4.88–9.70)	6.40 (4.70–9.80)	.940
cTNT (ng/mL)	0.036 (0.014–0.106)	0.037 (0.014–0.068)	0.033 (0.014–0.147)	.424
CK-MB (ng/mL)	2.21 (1.19–4.71)	1.95 (0.94–2.99)	2.46 (1.29–5.80)	.077
NT-proBNP (pg/mL)	1464 (458.25–4195)	1655.5 (394.4–9010.8)	1464 (471.5–4082)	.819
AKI stage				
0	47 (44.76)	11 (50.00)	36 (43.37)	.578
1	29 (27.62)	6 (27.27)	23 (27.71)	.967
2	11 (10.48)	3 (13.64)	8 (9.64)	.586
3	18 (17.14)	2 (9.10)	16 (19.28)	.260

* Statistically significant.

Continuous variables are expressed as mean ± standard deviation or median (interquartile range) according to normality. Categorical variables are expressed as frequency (percentages).

ECMO: extracorporeal membrane oxygenation; PCI: percutaneous coronary intervention; MI: myocardial infarction; COPD: chronic obstructive pulmonary disease; ALT: alanine aminotransferase; AST: aspartate aminotransferase; Ctnt: cardiac troponin T; CK-MB: creatinine kinase-myocardial band; NT-proBNP: N-terminal pro-B-type natriuretic peptide; AKI: acute kidney injury.

Surgical and mechanical support details of the patients are presented in Table 2. Eighty-six (81.90%) patients underwent cardiac surgery, and three (2.86%) patients underwent pulmonary surgery. Among the other procedures performed on the 16 patients were liver transplantation (*n* = 3), kidney transplantation (*n* = 2), hepatectomy (*n* = 1), Whipple procedure (*n* = 1), gastric ulcer rupture repair (*n* = 1), lumbar spine surgery (*n* = 2), resection of sacral malignancy (*n* = 1), thigh amputation (*n* = 1), hand trauma with pedicled flap grafting (*n* = 1), cesarean section (*n* = 1), total hysterectomy (*n* = 1) and abdominal wall debridement (*n* = 1). Seventy-seven (73.33%) patients received CCRT. The predominant mode of ECMO was veno-arterial ECMO (*n* = 97, 92.38%), and the remaining was veno-venous ECMO (*n* = 8, 7.62%). The main indication for ECMO was severe heart failure (*n* = 94, 89.52%), followed by severe pneumonia (*n* = 6, 5.71%) and interstitial pneumonia (*n* = 7, 6.67%). ECMO was initiated in 5 patients before surgery, 43 during surgery, and 57 after surgery. The mean time to initiate ECMO before surgery was 4.60 ± 4.93 d preoperatively, and the mean time to initiate ECMO after surgery was 6.27 ± 8.99 d postoperatively. ECMO was performed in the ICU if the patient initiated ECMO preoperatively and postoperatively, and acutely in the operating room if ECMO was initiated intraoperatively.

**Table 2.** Surgery and mechanical support of surgical patients receiving ECMO.

Variables	Total (*n* = 105)	Survival (*n* = 22)	Death (*n* = 83)	*p*-value
**Surgery**				
Cardiovascular surgery	86 (81.90)	20 (90.91)	66 (79.52)	.217
CABG	13 (12.38)	1 (4.55)	12 (14.46)	.209
Valve surgery	51 (48.57)	13 (59.09)	38 (45.78)	.267
Aortic surgery	17 (16.19)	3 (13.64)	14 (16.87)	.715
Congenital heart surgery	6 (5.71)	1 (4.55)	5 (6.02)	.791
Heart transplantation	8 (7.62)	1 (4.55)	7 (8.43)	.541
Lung surgery	3 (2.86)	0 (0.00)	3 (3.61)	.366
Others	16 (15.24)	2 (9.10)	14 (16.87)	.367
**Mechanical support**				
CRRT	77 (73.33)	13 (59.09)	64 (77.11)	.089
ECMO mode				
VA ECMO	97 (92.38)	22 (100)	75 (90.36)	.130
VV ECMO	8 (7.62)	0	8 (9.64)	
ECMO indication				
Severe heart failure	78 (72.29)	17 (77.27)	61 (73.49)	.718
Cardiac arrest	3 (2.86)	1 (4.55)	2 (2.41)	.593
Myocarditis	2 (2.41)	0	2 (2.41)	.462
Cardiac transplantation	10 (9.52)	2 (2.41)	8 (9.64)	.938
Severe pneumonia/ARDS	14 (13.33)	2 (2.41)	12 (14.46)	.510
Pulmonary infarction	7 (6.67)	0	7 (8.43)	.184
Lung transplantation	1 (0.95)	0	1 (0.95)	.605

Continuous variables are expressed as mean ± standard deviation or median (interquartile range) according to normality. Categorical variables are expressed as frequency (percentages).

ECMO: extracorporeal membrane oxygenation; CABG: coronary artery bypass graft; CRRT: continuous renal replacement therapy; VA ECMO: veno-arterial extracorporeal membrane oxygenation; VV ECMO: veno-venous extracorporeal membrane oxygenation.

* Statistically significant.

### Risk factors for mortality in surgical patients undergoing ECMO implantation

**Figure 2.**
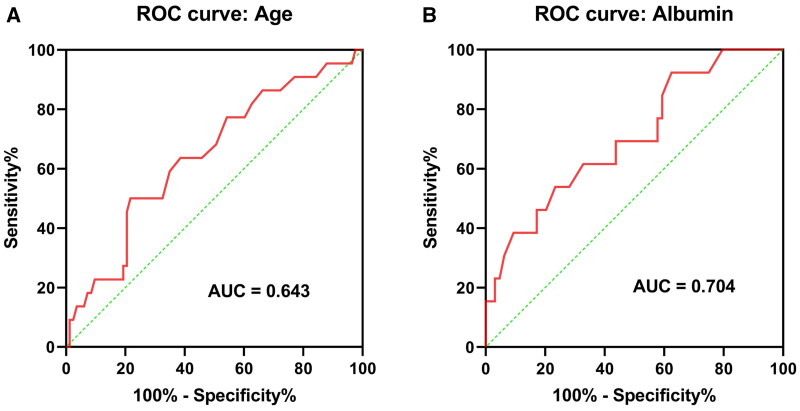
ROC curve analysis for predicting mortality in surgical patients. (A) ROC curve analysis of age for predicting death in surgical patients undergoing ECMO implantation. (B) ROC curve analysis of age for predicting death in surgical patients undergoing ECMO implantation. ROC: receiver operating characteristic; ECMO: extracorporeal membrane oxygenation.

A univariate logistic regression analysis was performed to identify the potential risk factors for mortality in surgical patients undergoing ECMO implantation. As shown in Table 3, the results showed that CRRT implantation, male, age, lymphocyte count, and albumin were associated with mortality. The multivariate logistic regression analysis showed that the independent risk factors affecting the prognosis after surgery were CRRT implantation (OR 3.385, 95% CI 1.058–10.827, *p* = .040), male sex (OR 0.174, 95% CI 0.040–0.750, *p* = .019), and age (OR 1.042 per year, 95% CI 1.004–1.082, *p* = .031). The ROC curve analysis showed that age 48.5 years had the highest Youden index (area under the curve [AUC] 0.643, 95% CI 0.514–0.773, *p* = .039; Figure 2(A)), and age >48.5 years had an OR of 4.440 (95% CI 1.520–12.970, *p* = .006).

**Table 3.** Univariate and multivariate analysis of risk factors for mortality in surgical patients undergoing ECMO implantation.

	Univariable		Multivariate	
Variables	OR (95% CI)	*p*-value	OR (95% CI)	*p*-value
CRRT	2.332 (0.865–6.290)	.094	3.385 (1.058–10.827)	.040*
Male	0.294 (0.080–1.077)	.065	0.174 (0.040–0.750)	.019*
Age (years)	1.034(1.000–1.069)	.051	1.042 (1.004–1.082)	.031*
Lymphocyte (×10^9/L)	0.531 (9,261–1.083)	.082	0.573 (0.251–1.308)	.186
Albumin (g/L)	0.915 (0.829–1.009)	.076	0.935 (0.842–1.037)	.202
APACHE II	1.034 (0.973–1.098)	.279	–	–
AKI	1.567 (0.609–4.030)	.352	–	–

*Statistically significant.

ECMO: extracorporeal membrane oxygenation; CRRT: continuous renal replacement therapy; OR: odds ratio; CI: confidence interval; APACHE II: Acute Physiology and Chronic Health Evaluation II; AKI: acute kidney injury.

### Risk factors for mortality in surgical patients with combined ECMO and CRRT

Because CRRT implantation significantly affects the prognosis of patients with ECMO, we further analyzed the independent risk factors for this category of patients with CRRT and ECMO implantation combined. Of the total 105 patients included in the final analysis, 77 (73.33%) were treated with CRRT (Figure 1). The univariate logistic regression analysis showed that age, valvular heart disease, and albumin were associated with mortality in surgical patients with ECMO implantation treated with CRRT. The multivariate logistic regression analysis showed that age (OR 1.064 per year, 95% CI 1.005–1.127, *p* = .034), valvular heart disease (OR 0.117, 95% CI 0.024–0.571, *p* = .008), and albumin (OR 0.804 per g/L, 95% CI 0.685–0.944, *p* = .008) remained the independent risk factors for prognosis (Table 4). Furthermore, the ROC curve analysis showed that albumin had the highest Youden index at a cutoff value of 39.95 g/L, with a specificity of 76.56% and a sensitivity of 53.85% for predicting death in surgical patients with both CRRT and ECMO implantation (AUC 0.704, 95% CI 0.548–0.859, *p* = .021; Figure 2(B)). When albumin <39.95 g/L was used as a risk factor, the OR in the multivariate logistic regression analysis was 5.539 (95% CI 1.339–22.921, *p* = .018).

**Table 4.** Univariate and multivariate analysis of risk factors for mortality in surgical patients with ECMO implantation treated with CRRT.

	Univariable		Multivariate	
Variables	OR (95% CI)	*p*-value	OR (95% CI)	*p*-value
Age (years)	1.036 (0.995–1.070)	.085	1.064 (1.005–1.127)	.034*
Valvular heart disease	0.284 (0.083–0.978)	.046*	0.117 (0.024–0.571)	.008*
Albumin (g/L)	0.841 (0.737–0.961)	.011*	0.804 (0.685–0.944)	.008*
APACHE II	1.037 (0.960–1.12)	.358	–	**-**
AKI	1.650 (0.181–2.334)	.509	–	**-**

* Statistically significant.

ECMO: extracorporeal membrane oxygenation; CRRT: continuous renal replacement therapy; OR: odds ratio; CI: confidence interval; APACHE II: Acute Physiology and Chronic Health Evaluation II; AKI: acute kidney injury.

## Discussion

In the present study, we found that CRRT implantation, male sex, and age were the independent risk factors for mortality in surgical patients undergoing ECMO implantation. In surgical patients on combined CRRT and ECMO, age, lack of valvular heart disease, and albumin were the independent risk factors for prognosis.

Patients receiving ECMO experience significant AKI, which is associated with a significant increase in mortality [19]. The patient’s primary diseases and the risk factors inherent to ECMO contribute to the development of AKI in ECMO patients. In addition, studies have shown that patients receiving ECMO have the highest risk of AKI prior to intubation, which is closely related to the severity and etiology of their primary disease, such as respiratory failure, heart failure, hypotension, cardiac arrest, ischemia, and nephrotoxin exposure [2,5]. Simultaneously, hemodynamic changes after ECMO cannulation affect renal blood flow *via* mechanisms including systemic inflammation, hemolysis, microcirculatory dysfunction, and platelet/coagulation abnormalities [1]. CRRT is increasingly used in ECMO patients to manage AKI and to prevent and treat fluid overload. Hemofiltration of blood removes some inflammatory proteins from the blood and has been proposed to exert an anti-inflammatory effect, though data to support the benefit of hemofiltration over other modalities of RRT in critically ill patients are lacking [20]. In 2010, the Kidney Interventions During Membrane Oxygenation study group surveyed 65 participating extracorporeal life support organization centers and showed that the most common indication for ECMO-initiated CRRT was fluid overload, which accounts for 43% [21]. In our study, the main indication of surgical patients who initiated ECMO was mainly heart failure, which accounted for 89.52%. In heart failure, fluid overload is usually a major clinical feature and problem that warrants correction. CRRT provide an easily initiated and efficient method of renal replacement and fluid management. And the distinction in the AKI stage between the groups of surviving and deceased patients is not of statistical significance. This may be attributed to the presence of multiple severe risk factors in surgical patients, where AKI alone isn’t sufficient to significantly elevate the mortality rate. Naturally, this doesn’t exclude the possibility that the limited sample size of this study contributed to the result.

Therefore, it was not surprising that we found that CRRT implantation was a significant independent risk factor for mortality in surgical patients with ECMO implantation, which was consistent with some studies [22,23]. On the other hand, some studies reported no significant difference in mortality between patients with ECMO implantation who received CRRT and those who did not [24–26]. A meta-analysis conducted by Han et al. in 2015 including 21,642 patients showed a higher mortality rate for ECMO patients requiring CRRT compared to ECMO alone, which is the current prevailing view [27]. In the same study, the authors concluded that the high mortality rate in patients receiving CRRT was not caused by CRRT per se, but by the severity of the original specific disease, in line with what we believe. In addition, they found an association between early initiation of CRRT and improved survival in patients who received ECMO and required CRRT [27]. Except for one study including pediatric patients after cardiac surgery that showed a higher mortality rate in the group of patients treated with CRRT [14], studies on CRRT in surgical patients implanted with ECMO are scarce. A study about children requiring CRRT and ECMO after cardiac surgery showed that early use of CRRT was associated with improved survival, with each additional day of CRRT initiation after ECMO associated with an approximately 2% increase in mortality [14]. These and other observational data suggest that CRRT, especially if applied relatively early in the course of illness, may benefit patients requiring ECMO, but high-quality data to support this view are lacking and some experts recommend applying the usual criteria for timing of RRT initiation in critical illness in patients on ECMO [28].

In this study, we found that age, lack of valvular disease, and albumin were significant risk factors for mortality in surgical patients on combined CRRT and ECMO. Higher age tends to indicate that the patient may have more underlying disease and a more fragile organism to compensate for. Consistent with this study, Thajudeen et al. found higher survival rates in younger patients with simultaneous ECMO and CRRT implantation [29]. However, it has also been suggested that age is not a risk factor for poor prognosis in patients with combined CRRT and ECMO [30]. This difference in findings between the former study and our study may be because the mean age in the former study was younger (survivors, 38.6 years; non-survivors, 42.7 years), whereas the mean age of the patients in our study was older (53.79 years), and advanced age was more detectable as a risk factor. Due to the irreversibility of age, age as a risk factor for mortality gives limited therapeutic guidance to clinicians but is suggestive for the assessment of poor patient prognosis. Interestingly, in our study, we found valvular disease to be a protective factor for poor prognosis. We speculate that patients with valvular disease have a better underlying condition and better postoperative recovery than patients who undergo other cardiac procedures, such as coronary artery bypass surgery, aortic surgery, and heart transplantation. However, the exact mechanism remains unclear. Albumin was a protective factor for patient mortality in this study, which is consist with a study including 114 adult patients receiving VA-ECMO [31]. In this study, we further calculated the optimal cutoff value of albumin as 39.95 g/L using ROC curve analysis though the overall predictive value was modest according to a series studies that an AUC of 0.7 is defined by some experts to be at the border between poor and acceptable discrimination [32]. Though statistically the strongest cutoff in our study, using a serum albumin of 39.95 g/dL, which is within the normal range, to make either prognostic or treatment decisions might not clinically appropriate. If albumin supplementation has any impact at all on prognosis in the setting of ECMO use, additional data would be needed to better define indications for use. However, low serum albumin may simply be a marker for poor prognosis rather than a truly modifiable risk factor. Apart from the potential for harm from albumin use in patients with traumatic brain injury, whether albumin supplementation is effective or not in critically ill patients remains controversial [33,34]. In a *post hoc* study of critically ill patients with traumatic brain injury, fluid resuscitation with albumin was associated with higher mortality rates than was resuscitation with saline [35]. Further studies are needed to determine whether albumin infusion is significantly associated with the prognosis of patients receiving ECMO. Additionally, we discovered that in the univariate analysis, the APACHE II score is not a mortality risk factor for this cohort. This might be because the APACHE II scoring system was developed before the widespread use of ECMO and has not been validated in this patient group [36]. In a single-center study published by Ng et al. they found that in patients undergoing VA-ECMO, APACHE II tends to underestimate the mortality rate of low-risk patients and overestimate it for high-risk ones [37]. Most patients in our study underwent cardiac surgery, and existing research indicates that APACHE II is less accurate than other models in predicting in-hospital mortality for cardiac surgery patients [38,39].

This study has several limitations. First, this was a retrospective single-center study and inherent limitations exist including the risk of unrecognized bias, the risk of residual confounding, and limited generalizability to other centers or centers in other nations with different patient populations and different practice patterns. Second, the test results included in this study were those of the patient’s last routine preoperative blood draw and do not fully demonstrate the patient’s final preoperative status. And since the majority of patients in this study underwent VA-ECMO, the findings may not apply to patients requiring VV-ECMO. Moreover, the majority of surgical patients included in this study were cardiac surgery patients, and only a small number of patients underwent pulmonary or other surgeries. Further expansion of the sample size of patients undergoing other surgeries is needed to further broaden the main findings of this study. Furthermore, the time and the way of ECMO implantation were not included in the analysis and may affect the results. The temporal relationship between ECMO initiation and surgery, the start context of ECMO, the kind of ECMO cannulation may have some influence on the risk of mortality, but the objective of our study is to estimate the risk and provide some guidance for treatment. Further multicenter study and larger sample size were needed.

In summary, we found that CRRT implantation was an independent risk factor for surgical patients with ECMO implantation, in addition to age and sex. The mortality rate was higher in surgical patients undergoing concomitant CRRT and ECMO treatment. In this group of patients, higher age, non-valvular disease, and low albumin were independent risk factors for mortality. Albumin had the highest Youden index at a cutoff value of 39.95 g/L for predicting mortality, though the overall predictive value was modest (area under ROC 0.704). Age had the highest Youden index at a cutoff value of 48.5 years for predicting mortality.

## Data Availability

The datasets used and/or analyzed during the current study are available from the corresponding author on reasonable request.
